# Editorial: Genomics-based strategies for advanced drug resistance and epidemiological surveillance of *Mycobacterium tuberculosis* and other non-tuberculous mycobacteria

**DOI:** 10.3389/fmicb.2023.1264285

**Published:** 2023-08-23

**Authors:** Arash Ghodousi, Davood Darban-Sarokhalil, Abdolrazagh Hashemi Shahraki, Sivakumar Shanmugam

**Affiliations:** ^1^Division of Immunology, Transplantation and Infectious Diseases, Vita-Salute San Raffaele University, Milan, Italy; ^2^Department of Microbiology, School of Medicine, Iran University of Medical Sciences, Tehran, Iran; ^3^College of Medicine, University of Florida, Gainesville, FL, United States; ^4^National Institute of Research in Tuberculosis (ICMR), Chennai, India

**Keywords:** *Mycobacterium tuberculosis*, non-tuberculous mycobacteria, genomics, next generation sequencing (NGS), antibiotic resistance, epidemiological surveillance

In the pursuit of advancing diagnostics and eradicating mycobacterial infections, recent studies have unveiled promising methodologies that hold tremendous potential for revolutionizing our approach to detecting and managing tuberculosis (TB) and non-tuberculous mycobacteria (NTM) infections. The integration of Next-Generation Sequencing (NGS) techniques has emerged as a powerful tool in this endeavor, allowing for real-time, comprehensive, and sensitive data generation for disease outbreak investigations and surveillance programs.

One such study by Zeineldin et al. explores the direct sequencing of *Mycobacterium bovis* (*M. bovis*) DNA from tissue samples using the SureSelect target enrichment method. The researchers demonstrate that this approach effectively reduces host DNA, enabling successful sequencing of low to mid-titer samples. The genomes of *M. bovis* are successfully sequenced, allowing for strain classification and contact tracing, critical for the swift containment of bovine tuberculosis (bTB) outbreaks. While SNP inconsistencies between directly sequenced genomes and cultured isolates are observed, this method still holds promise as a routine diagnostic procedure to support bTB surveillance and eradication programs.

Another study by He et al. focuses on the recurrence of TB cases in Hunan province, China, shedding light on the importance of extended post-treatment follow-up. Through genomic analysis of recurrent TB patients, the researchers identify the main cause of recurrence as endogenous relapse. This finding underscores the need for vigilant management of TB patients post-treatment. Additionally, the high frequency of fluoroquinolone resistance in relapsed TB cases raises awareness about cautious antibiotic selection, emphasizing the importance of drug susceptibility testing (DST) results in guiding treatment decisions.

In the quest for optimized mycobacterial DNA extraction protocols, Prajwal et al. explore various methods to enhance diagnostic yield. The use of saponin and “DNase I” is found to effectively deplete human DNA while slightly increasing mycobacterial DNA yield, particularly beneficial for molecular diagnostics with low mycobacterial loads. By avoiding decontamination and selecting appropriate automated nucleic acid extraction processes, the reliability and sensitivity of mycobacteria molecular diagnostics can be enhanced, thereby enabling more precise diagnoses and treatments. In the pursuit of improving molecular testing for mycobacterial infections, a separate study by Kok et al. compares different extraction devices and introduces the innovative Saponin-Mycobacteria-genome sequencing (SMg) protocol. The study concludes that SMg effectively depleted human DNA while preserving mycobacterial DNA, offering increased sensitivity for detecting low mycobacterial loads.

Moreover, the comparison of short-read sequencing (SRS), long-read sequencing (LRS), and a hybrid approach (HYBR) for *Mycobacterium tuberculosis complex* (MTBC) genome analysis by Di Marco et al. reveals the HYBR approach as the most promising option. With its comprehensive view and enhanced resolution in variant calling and *de novo* assembly, the HYBR approach offers researchers a powerful tool to delve deeper into the study of MTBC genomes.

In addition, the diagnostic efficacy of the MassARRAY system in detecting drug-resistant TB is evaluated by Yang et al.. According to the authors, the high accuracy and sensitivity of this system make it a promising technology for diagnosing drug-resistant TB, aiding treatment decisions, and supporting specialized TB hospitals.

Lastly, in the context of DST for NTM, Solanki et al. review the latest research on the potential of whole-genome sequencing (WGS) to predict drug resistance. WGS's genotypic approach offers a way to predict drug susceptibility, overcoming limitations associated with traditional phenotypic methods. However, challenges remain in implementing WGS for NTM DST, necessitating further research and the development of accurate databases.

As we reflect on these studies collectively, a clear path emerges toward a future of precision diagnostics and targeted eradication strategies. NGS technologies present unparalleled opportunities to improve our understanding of mycobacterial infections and offer real-time data to support swift action in outbreak scenarios. These advancements hold the potential to transform our approach to diagnosing and treating mycobacterial infections, furthering our progress toward TB eradication goals.

While the potential is promising, the journey is not without its challenges. Ensuring accessibility to advanced equipment and establishing accurate databases for predicting resistance based on WGS data will be critical. Larger studies will be necessary to validate the performance of these techniques on different sample types with low microbial loads. Collaboration between researchers, clinicians, and policymakers will be instrumental in overcoming these hurdles and bringing these cutting-edge technologies to the forefront of clinical practice.

In conclusion, these studies underscore the transformative potential of NGS techniques in the fight against mycobacterial infections. As we harness the power of genomic analysis, we step closer to achieving precision diagnostics, enhancing surveillance programs, and ultimately realizing the dream of a TB-free world. Continued research, collaboration, and dedication to innovation will be the driving forces that carry us through the threshold of success in the battle against mycobacterial infections.

## Author contributions

AG: Conceptualization, Writing—original draft, Writing—review, and editing. DD-S, AS, and SS: Writing—review and editing.

